# Impact of Comorbid Chronic Conditions to Quality of Life among Elderly Patients with Diabetes Mellitus in Vietnam

**DOI:** 10.3390/ijerph16040531

**Published:** 2019-02-13

**Authors:** Huong Van Nguyen, Tung Thanh Tran, Cuong Tat Nguyen, Tung Hoang Tran, Bach Xuan Tran, Carl A. Latkin, Cyrus S.H. Ho, Roger C.M. Ho

**Affiliations:** 1 Department of Neuroscience, Hanoi Medical University, Hanoi 100000, Vietnam; vanhuong73@hotmail.com; 2 Center of Excellence in Evidence-based Medicine, Nguyen Tat Thanh University, Ho Chi Minh 70000, Vietnam; tung.coentt@gmail.com; 3 Institute for Global Health Innovations, Duy Tan University, Da Nang 550000, Vietnam; cuong.ighi@gmail.com; 4 Department of Lower Limb Surgery, Vietnam-Germany Hospital, Hanoi 100000, Vietnam; tranhoangtungvdh@gmail.com; 5 Institute for Preventive Medicine and Public Health, Hanoi Medical University, Hanoi 100000, Vietnam; 6 Johns Hopkins Bloomberg School of Public Health, Baltimore, MD 21205, USA; carl.latkin@jhu.edu; 7 Department of Psychological Medicine, National University Hospital, Singapore 119074, Singapore; cyrushosh@gmail.com; 8 Department of Psychological Medicine, Yong Loo Lin School of Medicine, National University of Singapore, Singapore 119077, Singapore; pcmrhcm@nus.edu.sg; 9 Center of Excellence in Behavior Medicine, Nguyen Tat Thanh University, Ho Chi Minh 70000, Vietnam

**Keywords:** diabetes, type 2 diabetes mellitus, quality of life, elderly, comorbidities

## Abstract

Type 2 diabetes mellitus (T2DM) is a major cause of disease burden in the elderly population. This study aimed to measure the quality of life (QOL) among patients with T2DM and the associations between co-morbidities and QOL. A cross-sectional study was conducted on 194 patients with T2DM. The minimal clinically important difference (MCID) scores were used to indicate the clinically meaningful differences of comorbidities on quality of life. A Tobit regression was employed to find relationships between QOL and comorbidities. The mean scores of QOL domains were 50.8 (*SD* = 13.2) in physical, 62.4 (*SD* = 11.5) in psychological, 52.3 (*SD* = 10.2) in social relationship, and 64.3 (*SD* = 10.1) in environmental. Digestive and neuropsychiatric diseases had the strongest negative associations with physical QOL of patients. Neuropsychiatric diseases also had the biggest effect on psychological and environmental QOL. Meanwhile, in the social domain, respiratory diseases had the greatest effect. In conclusion, patients with T2DM struggled to perform physical functions. In addition, comorbidities significantly reduced the QOL of T2DM patients.

## 1. Introduction

Type 2 diabetes mellitus (T2DM) has been acknowledged as one of the most serious public health problems worldwide [1]. According to the World Health Organization (WHO), the prevalence of T2DM in the adult population increased from 4.7% in 1980 to 8.5% in 2014, with approximately 422 million patients [1]. Additionally, T2DM is a major cause of disease burden in the elderly population [2]. This has been a noteworthy problem since the life expectancy has been increasing recently [3]. Elderly people with T2DM also are more likely to have co-comorbidities, such as cardiovascular diseases, neuropsychiatric diseases, and respiratory diseases [3].

In Vietnam, approximately 3.5 million Vietnamese adults suffer from T2DM [4]. Data from two national surveys indicated that the prevalence of T2DM in the Vietnamese population increased from 2.7% in 2002 to 5.4% in 2012 [5]. Meanwhile, the aging population in Vietnam is significant [6]. Results from a previous study found a positive association between age and the occurrence of T2DM [5]. Thus, measuring the well-being of elderly people with T2DM is necessary to draw further implications for healthcare system.

Quality of life (QOL) is considered an indicator of the well-being and health care needs among elderly populations [7]. Many scales have been developed to measure the QOL concept [8,9,10]. The World Health Organization Quality of Life-Brief scale (WHOQOL-BREF) has been acknowledged as being a comprehensive scale to assess the QOL [11]. WHOQOL-BREF is also commonly used to assess the QOL among elderly people and patients with chronic diseases such as diabetes [11].

The co-existence of comorbidities shows a negative influence on the well-being of elderly people with T2DM [12,13,14,15,16]. Previous research indicated an association between chronic diseases and impaired QOL [17], as well as psychological problems of patients with T2DM [12,13]. In addition, elderly patients with T2DM have more difficulties in maintaining their usual social relationships [15]. To measure the clinically meaningful differences of comorbidities on QOL, many researchers have used the minimal clinically important difference (MCID) indicator [18,19,20]. It is a useful measurement for assessing the impact of diseases on the QOL of patients [18,19,20].

Many studies in Vietnam have measured the QOL of patients living with T2DM. Results from those studies found a lower QOL in patients living with T2DM compared to the general population [5,21]. In addition, the studies found associations between the QOL and socioeconomic characteristics of patients living with T2DM [21]. However, none of these studies investigated the correlations between the co-existence of comorbidities and QOL of T2DM patients. Thus, this study aimed to measure the QOL and the associations between co-morbidities and QOL among patients with T2DM.

## 2. Materials and Methods

### 2.1. Study Setting

A cross-sectional study was conducted from February to September 2016 at the Department of Endocrinology and Metabolism and the Outpatient Department of National Geriatric Hospital. Participants were recruited using a convenient sampling approach. Patients who met the following criteria were invited to participate in the study: (1) 60 years old or older; (2) have been diagnosed with T2DM according to WHO’s diabetes criteria 2006 [22]; (3) receiving treatments at the National Geriatric hospital; (4) have the ability to answer the interviewer. Patients were excluded from the study if they were having kidney failure or using drugs that caused peripheral neuropathy such as vincristine or metronidazole. A total of 194 patients agreed to participate in the study.

### 2.2. Measurements and Instruments

Face-to-face interviews using a structured questionnaire were used to collect patients’ data. Each interview lasted 10–15 min. To avoid social desirability bias, we invited patients to a private room and did not recruit healthcare workers for collecting data. Interviewers were students who were enrolled in medical and nursing programs at Hanoi Medical University. They were trained by professionals to ensure the quality of data. The data collected are described below.

#### 2.2.1. Demographics Characteristics

We collected data about gender, employment, living area, education, marital status, smoking and alcohol consumption, current treatment regime, and age.

#### 2.2.2. Health Status

Health status data were imported from participants’ medical records after asking for their consent. Information included their height (cm), weight (kg), T2DM duration (years), cholesterol (mmol/L), triglyceride (mmol/L), High-Density Lipoprotein cholesterol (HDL-c) (mmol/L), Low-Density Lipoprotein cholesterol LDL-c (mmol/L), glucose level (mmol/L), HbA1C (%), and comorbidities. We calculated Body Mass Index (BMI) by the formula: BMI index = weight ÷ height^2^. Then, based on the WHO guideline, we categorized patients into three groups: low weight, normal weight, and overweight [23].

#### 2.2.3. Dyslipidemia

ATP III Guidelines was used to diagnose dyslipidemia. Patients were diagnosed as experiencing dyslipidemia if they had cholesterol levels > 5.2 mmol/L, triglyceride > 1.73 mmol/L, HDL-c < 0.9 mmol/L, and LDL-c > 3.4 mmol/L [24].

#### 2.2.4. Hypertension

According to “The Seventh Report of the Joint National Committee on Prevention, Detection, Evaluation, and Treatment of High Blood Pressure”, patients had hypertension if their diastolic blood pressure ≥ 90 mmHg and/or systolic blood pressure ≥ 140 mmHg [25].

#### 2.2.5. Quality of Life

To measure QOL, we used WHOQOL-BREF, which has been used elsewhere to evaluate QOL of T2DM patients [26]. WHOQOL-BREF includes 26 questions which can be divided into four domains, including physical, psychological, social relationship, and environmental [27]. The score for each question ranges from 1 (very poor QOL) to 5 (very good QOL). The raw score of each domain was calculated by summing the scores of all of the questions in the domain. The raw scores vary across domains: 7–35 in physical; 6–30 in psychological; 3–15 in social relationship, and 8–40 in environmental. Next, the raw score was transformed to a 100-point scale according to WHO guidelines, in which a higher score means a higher QOL of T2DM patients [28].

### 2.3. Statistical Analysis

Data were analyzed using STATA 15.0 (Stata Corp. LP, College Station, TX, USA). Frequency (%) was used to present categorical variables. Quantitative variables were reported using mean, standard deviation (*SD*) or median, and interquartile range (IQR). MCID was calculated to illustrate the clinically meaningful or important difference in QOL domains among patients with or without comorbidities. Univariate and multivariate Tobit regressions were employed to find correlations between QOL and comorbidities. A *p*-value < 0.05 was considered statistically significant.

### 2.4. Ethics Approval

The study protocol was approved by the Institutional Review Board of Hanoi Medical University. The ethical code is 01/HMUIRB.

## 3. Results

Most of the participants were female (62.4%), white collar workers (56.7%), lived in the urban area (82.5%), had high school education or higher (57.2%), and were married (67.0%). The percentage of patients who never smoked or drank alcohol were 72.7% and 74.7%, respectively (Table 1).

Table 2 showed that there were 60.8% patients suffering T2DM for 10 years or less. Most of the patients had dyslipidemia (74.2%) or hypertension (71.1%). More than half of patients did not have comorbidities.

Table 3 indicates that the mean score of the QOL domains were as follows: 50.8 (*SD* = 13.2) in physical, 62.4 (*SD* = 11.5) in psychological, 52.3 (*SD* = 10.2) in social relationship, and 64.3 (*SD* = 10.1) in environmental. The QOL score was higher in males and in patients who had high school education or higher. There were no significant differences among employment, living area, and marital status groups.

**Figure 1:**
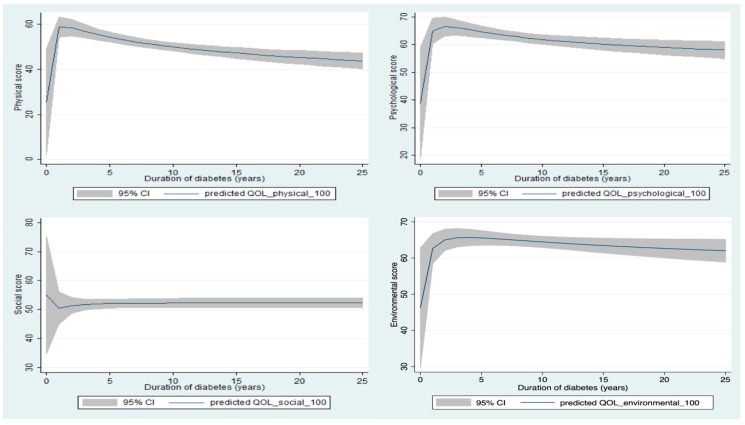
Correlations between diabetes duration and WHOQOL-BREF domain scores. QOL: quality of life.

Figure 1 illustrates the correlations between T2DM duration and the QOL of patients. The physical, psychological, and environmental QOL scores increased in the first few years after being diagnosed with T2DM. Then, they decreased in the following years. In contrast, the social QOL decreased in the early stage then increased after that.

**Figure 2:**
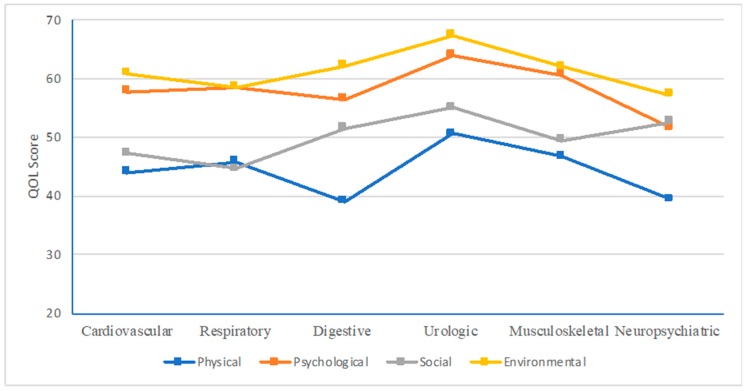
WHOQOL-BREF domain scores of respondents with health problems.

Figure 2 shows that patients with neuropsychiatric diseases had the lowest QOL scores in three domains, including the physical, psychological, and environmental domains. Meanwhile, patients with respiratory diseases had the lowest score in the social relationship domain.

Table 4 showed that there were significant MCIDs in the physical and psychological domains between patients with and without cardiovascular, digestive, and neuropsychiatric diseases. In the social domain, cardiovascular and musculoskeletal diseases were found to have significant MCIDs. The number of diseases also had a significant negative impact on QOL in the physical, social, and environmental domains.

Table 5 shows that the number of diseases had a negative association with the QOL of elderly people with T2DM. When using univariate Tobit regression, we found that the score of the physical domain was affected mostly by the digestive diseases (Coef. = −12.77, 95% CI = −19.32; −6.21) and neuropsychiatric diseases (Coef. = −11.84; 95% CI = −21.73; −1.95). Neuropsychiatric diseases had the largest effect on psychological (Coef. = 11.13, 95% CI = −19.69l; −2.57) and environmental domains (Coef. = −9.55; 95% CI = −16.90; −2.21). In the social domain, respiratory diseases had the most negative effect (Coef. = −7.84; 95% CI = −16.07; 0.38). Similar results were found in the adjusted model.

## 4. Discussion

This study indicated that QOL scores of T2DM elderly patients were at the moderate level in four domains. The physical domain was the domain that had the lowest score. Patients with T2DM were reported to have higher QOL scores in physical, psychological, and environmental domains in the early years of diabetes, while the opposite trend was found in the social domain. By using MCID, we found the clinically meaningful difference of comorbidities on QOL. Respiratory diseases had the most negative effect on the social relationship domain. Meanwhile, in the physical domain, digestive and neuropsychiatric diseases had the strongest negative associations with QOL score. Moreover, patients with co-existence of T2DM and neuropsychiatric diseases also had the strongest correlations with the score of psychological and environmental domains.

By using MCID, significant differences in QOL between patients with or without comorbidities were found in this study. The MCID may help to represent the numeric value of clinical change of QOL. It is commonly used in longitudinal studies [18,19,20]. However, due to a lack of resources and budget, we were only able to conduct a cross-sectional survey to capture QOL at a time point. This approach has been used in a previous study [29]. The findings of this study supplement the available evidence regarding the usage of MCID in measuring QOL among patients with specific diseases in Vietnam.

QOL scores in four domains were at the moderate level, which had also been previously found in other research on T2DM patients [26,30]. Among the four domains, the physical domain had the lowest QOL score, which was in line with Chew et al.’s study in Malaysia [26]. This indicated that T2DM patients were having problems in performing their usual activities, including mobility and work-related issues. It also indicated that patients who participated in this study experienced physical pain, discomfort, and sleep problems [28]. These problems were also reported as the most common health problems of T2DM patients [30]. T2DM complications including cardiovascular diseases, food ulcers, or diabetic eye diseases [31] may have been some of the causes of the low score in this domain. Since many studies have also found that patients with T2DM may struggle with performing physical activities [26,30], it is important to offer more supports to T2DM patients for their physical activities and pain management in order to improve their QOL.

We found that digestive and neuropsychiatric diseases had the strongest correlations with the physical domain. We also found that T2DM patients with neuropsychiatric diseases experienced a lower QOL in psychological and environmental domains. T2DM patients are more likely to have more negative feelings and mental health problems [28]. Patients with neuropsychiatric diseases also felt less safe and less happy with their health and social care [28]. The negative effects of neuropsychiatric diseases on T2DM patients’ QOL have been investigated in previous studies, which indicated that the co-existence of diabetes and neuropsychiatric diseases had impaired the patients’ QOL, especially in terms of psychological aspects [12,13,14]. Another study found that T2DM was associated with anxiety and depression [32]. In addition, there is evidence of increasing health care use and expenditures in T2DM patients with neuropsychiatric comorbidities [33], which could explain our results that patients suffered problems in regard to healthcare costs and accessibility [28]. A strong correlation between digestive diseases and physical functions of T2DM patients was found in this study. However, there is a lack of evidence of this in the literature, suggesting further research is needed to fill this gap.

Respiratory diseases were found to have a negative association with the social relationship domain, which was consistent with prior studies [15,16,34]. The reason for this might be due to the fact that some common symptoms of respiratory diseases, such as coughing and wheezing, might limit the elderly patients from maintaining their usual social relationships [15]. Of note, George et al. found a higher risk of having respiratory diseases in T2DM patients [35]. These diseases in the elderly also require more healthcare resources than younger patients [36]. Therefore, more attention and social support from health workers and family are needed for T2DM elderly patients with respiratory diseases.

The results of this study suggest some implications. First, since the physical domain had the lowest score, more supports should be provided to T2DM patients to help them perform physical activities, reduce their pain, and improve their sleep quality of patients, thereby increasing their QOL. Second, the substantial impacts of comorbidities on the QOL of T2DM patients suggest that more attention should be paid to these patient groups. Finally, since the evidence provided by this study regarding the associations between the QOL and comorbidities of T2DM patients is limited to a Vietnamese context, further studies using WHOQOL-BREF and MCID are recommended to elaborate upon this study’s results.

Several limitations in the current study should be acknowledged. This is a cross-sectional study, therefore it is unable to determine the causal relationships between the changes of QOL and different comorbidities among T2DM patients. In addition, although MCID can be used in a cross-sectional study, the result may not reflect the actual MCID of QOL by different comorbidities. Further longitudinal studies are warranted to confirm these differences. Lastly, this study was conducted in a health facility and applied the convenient sampling method, which may limit the generalizability of the results.

## 5. Conclusions

In conclusion, our findings indicated that patients with T2DM had a moderate QOL level. T2DM elderly patients experienced most problem in the form of physical functions. Digestive and neuropsychiatric diseases had the strongest negative associations with the physical score. The existence of neuropsychiatric diseases also had the largest negative correlation with the QOL score in psychological and environmental domains. Meanwhile, respiratory diseases had the largest negative effect on the social relationship domain score. These results suggest more attention should be provided by the healthcare system toward elderly patients with T2DM in order to alleviate the effect of comorbidities on their QOL. Further studies on the correlations between comorbidities and QOL are recommended.

## Figures and Tables

**Table 1:** Demographic characteristics of respondents.

Characteristic	Female		Male		Total		*p*-Value
	*n*	%	*n*	%	*n*	%	
Total	121	62.4	73	37.6	194	100	-
Employment							
White collar	62	51.2	48	65.8	110	56.7	0.05
Blue collar	59	48.8	25	34.2	84	43.3	
Living area							
Urban	103	85.1	57	78.1	160	82.5	0.21
Rural	18	14.9	16	21.9	34	17.5	
Education							
Under high school	60	49.6	23	31.5	83	42.8	0.01
High school or above	61	50.4	50	68.5	111	57.2	
Marital status							
Married	81	66.9	49	67.1	130	67.0	0.98
Widow/Divorced	40	33.1	24	32.9	64	33.0	
Smoking status							
Never smoked	116	95.9	25	34.2	141	72.7	<0.01
Former smokers	4	3.3	35	47.9	39	20.1	
Current smokers	1	0.8	13	17.8	14	7.2	
Alcohol consumption							
Never drank	118	97.5	27	37.0	145	74.7	<0.01
Former drinker	2	1.7	27	37.0	29	14.9	
Current drinker	1	0.8	19	26.0	20	10.3	
Treatment							
Insulin	34	28.1	39	53.4	73	37.6	<0.01
Others	87	71.9	34	46.6	121	62.4	
	Mean	*SD*	Mean	*SD*	Mean	*SD*	
Age	71.2	7.0	70.4	6.5	70.9	6.8	0.48

**Table 2:** Health characteristic of elderly patients with type 2 diabetes mellitus (T2DM).

Characteristic	Female		Male		Total		*p*-Value
	*n*	%	*n*	%	*n*	%	
BMI category
Low weight	5	4.1	1	1.4	6	3.1	0.46
Normal weight	96	79.3	62	84.9	158	81.4
Overweight	20	16.5	10	13.7	30	15.5	
Diabetes duration
10 years or less	75	62.0	43	58.9	118	60.8	0.67
More than 10 years	46	38.0	30	41.1	76	39.2	
Dyslipidemia	93	76.9	51	69.9	144	74.2	0.28
Hypertension	85	70.2	53	72.6	138	71.1	0.73
Good glycemic control	55	45.5	21	28.8	76	39.2	0.02
Comorbidities
Cardiovascular	18	14.9	8	11.0	26	13.4	0.44
Respiratory	3	2.5	3	4.1	6	3.1	0.53
Digestive	10	8.3	6	8.2	16	8.2	0.99
Urologic	7	5.8	8	11.0	15	7.7	0.19
Musculoskeletal	32	26.4	13	17.8	45	23.2	0.17
Neuropsychiatric	3	2.5	4	5.5	7	3.6	0.28
Number of diseases
0	69	57.0	42	57.5	111	57.2	0.96
1	34	28.1	21	28.8	55	28.4
2	15	12.4	9	12.3	24	12.4
3	3	2.5	1	1.4	4	2.1	
	Median	IQR	Median	IQR	Median	IQR
Diabetes duration	9	6–12	9	4–15	9	5–13	0.48

**Table 3:** Differences in the World Health Organization Quality of Life-Brief scale (WHOQOL-BREF) domain scores between demographics characteristic.

Characteristic	Physical		*p*-Value	Psychological		*p*-Value	Social Relationship		*p*-Value	Environmental		*p*-Value
	Mean	*SD*		Mean	*SD*		Mean	*SD*		Mean	*SD*	
Total	50.8	13.2		62.4	11.5		52.3	10.2		64.3	10.1	
Gender												
Female	49.4	13.7	0.07	60.2	12	<0.01	51.7	11	0.98	63.1	10.7	0.03
Male	53.2	12.2		66.1	10		53.1	8.7		66.3	8.8	
Employment												
White collar	51.8	12.7	0.20	64.7	11	<0.01	52.3	11	0.69	66.5	9.5	<0.01
Blue Collar	49.6	13.9		59.5	12		52.2	9.7		61.4	10.3	
Living area												
Urban	50.5	12.9	0.54	62.3	12	0.99	52.4	10	0.70	64.5	10.3	0.67
Rural	52.5	14.7		63	9.5		51.8	9.4		63.4	9.6	
Education												
Under high school	49.6	14	0.23	59.4	12	<0.01	52.2	9.8	0.76	61.3	10.3	<0.01
High school or above	51.7	12.7		64.7	11		52.3	11		66.5	9.4	
Marital status												
Married	50.6	13	0.80	62.6	11	0.84	52.6	9.9	0.12	64	10.6	0.40
Widow/Divorced	51.3	13.9		62.1	12		51.7	11		64.9	9.2	
Treatment												
Insulin	47.2	13.3	0.01	61.4	11	0.19	53.6	8.8	0.19	64.6	10.7	0.54
Others	53	12.8		63.1	12		51.5	11		63.7	9.3	

**Table 4:** Between-group minimal clinically important difference (MCID) of WHOQOL-BREF domains scores for different comorbidities.

Characteristic	*n*	Physical		Psychological		Social		Environmental	
		Diff ^†^	95% CI	Diff ^†^	95% CI	Diff ^†^	95% CI	Diff ^†^	95% CI
Health problem									
No disease	111	-		-		-		-	
Cardiovascular	26	−7.9 *	−13.3; −2.5	−5.4 *	−10.1; −0.7	−5.8 *	−10.0; −1.7	−4.0	−8.2; 0.2
Respiratory	6	−5.2	−16.0; 5.7	−4.1	−13.5; 5.3	−7.8	−16.1; 0.4	−6.0	−14.3; 2.3
Digestive	16	−12.8 *	−19.4; −6.2	−6.5 *	−12.4; −0.7	−0.8	−6.0; 4.5	−2.2	−7.5; 3.0
Urologic	15	−0.3	−7.3; 6.8	1.7	−4.4; 7.8	3.0	−2.4; 8.4	3.4	−2.0; 8.7
Musculoskeletal	45	−5.3	−9.7; 0.9	−2.3	−6.1; 1.6	−3.6 *	−7.0; -0.2	−2.9	−6.3; 0.5
Neuropsychiatric	7	−11.8 *	−21.8; −1.9	−11.1*	−19.7; −2.5	0.3	−7.4; 8.1	−7.3	−14.9; 0.4
Number of comorbidities									
0	111	-		-		-		-	
1	55	−4.9	−10.2; 0.5	−1.4	−6.2; 3.3	−0.9	−5.1; 3.4	−1.7	−6.0; 2.6
2	24	−8.9 *	−16.2; −1.7	−4.8	−11.4; 1.7	3.3	−9.2; 2.5	−2.6	−8.5; 3.2
3	4	−25.9 *	−45.4; −9.4	−18.3	−33.1; −3.6	−14.2 *	−27.4; −1.0	−13.6 *	−26.8; −0.4

^†^ Difference between respondents with and without health conditions/diseases. * *p* < 0.05.

**Table 5:** Correlations between WHOQOL-BREF domain scores and comorbidities.

Characteristic	Physical		Psychological		Social Relationship		Environmental	
	Coef. (95% CI) ^a^	Coef. (95% CI) ^b^	Coef. (95% CI) ^a^	Coef. (95% CI) ^b^	Coef. (95% CI) ^a^	Coef. (95% CI) ^b^	Coef. (95% CI) ^a^	Coef. (95% CI) ^b^
Health problem								
Cardiovascular	−7.85 (−13.23; −2.47) *	−7.66 (−12.99; −2.32) *	−5.40 (−10.10; −0.69) *	−5.22 (−9.71; −0.74) *	−5.82 (−9.95; −1.68) *	−5.85 (−10.00; −1.71) *	−3.98 (−8.14; 0.19)	−3.81 (−7.84; 0.21)
Respiratory	−5.17 (−15.95; 5.61)	−5.21 (−15.90; 5.48)	−4.07 (−13.43; 5.29)	−4.33 (−13.25; 4.59)	−7.84 (−16.07; 0.38)	−8.29 (−16.53; −0.05) *	−5.97 (−14.21; 2.26)	−6.15 (−14.09; 1.80)
Digestive diseases	−12.77 (−19.32; −6.21) *	−12.78 (−19.27; −6.29) *	−6.55 (−12.37; −0.72) *	−6.09 (−11.65; −0.53) *	−0.77 (−5.99; 4.45)	−0.80 (−6.03; 4.44)	−2.22 (7.42; 2.98)	−1.46 (−6.49; 3.56)
Urologic diseases	−0.26 (−7.26; 6.74)	−0.88 (−7.90; 6.14)	1.69 (−4.38; 7.76)	−0.09 (−5.94; 5.77)	2.96 (−2.40; 8.32)	2.57 (−2.86; 8.01)	3.37 (−1.97; 8.72)	2.12 (−3.11; 7.34)
Musculoskeletal disorders	−5.29 (−9.66; −0.92) *	−4.91 (−9.27; −0.54) *	−2.28 (−6.12; 1.55)	−1.56 (−5.24; 2.13)	−3.59 (−6.96; −0.22) *	−3.46 (−6.86; −0.07) *	−2.86 (−6.24; 0.51)	−2.68 (−5.95; 0.60)
Neuropsychiatric diseases	−11.84 (−21.73; −1.95) *	−13.02 (−22.86; −3.18) *	−11.13 (−19.69; −2.57) *	−13.84 (−21.97; −5.72) *	0.31 (−7.39; 8.02)	−0.16 (−7.94; 7.62)	−7.27 (−14.88; 0.35)	−9.55 (−16.90; −2.21) *
Number of diseases								
0	REF	REF	REF	REF	REF	REF	REF	REF
1	−4.88 (−8.91; −0.86) *	−4.45 (−8.47; −0.44) *	−1.45 (−5.05; 2.16)	−0.93 (−4.37; 2.52)	−0.85 (−4.07; 2.37)	−0.99 (−4.24; 2.27)	−1.71 (−4.93; 1.52)	−1.21 (−4.33; 1.91)
2	−8.95 (−14.44; −3.45) *	−8.95 (−14.39; −3.51) *	−4.83 (−9.75; 0.09)	−5.24 (−9.90; −0.58) *	−3.34 (−7.74; 1.06)	−3.49 (−7.89; 0.92)	−2.63 (−7.03; 1.77)	−3.06 (−7.29; 1.16)
3	−25.86 (−38.29; −13.44) *	−25.98 (−38.26; −13.70) *	−18.33 (−29.46; −7.20) *	−18.46 (−28.98; −7.93) *	−14.22 (−24.16; −4.27) *	−13.91 (−23.86; −3.97) *	−13.63 (−23.57; −3.68) *	−14.17 (−23.71; −4.63) *
Multi-morbidity (≥2 diseases)								
No	REF	REF	REF	REF	REF	REF	REF	REF
Yes	−9.75 (−14.89; −4.61) *	−9.94 (−15.02; −4.85) *	−6.28 (−10.81; −1.75) *	−6.82 (−11.12; −2.53) *	−4.61 (−8.65; −0.58) *	−4.65 (−8.70; −0.61) *	−3.63 (−7.68; 0.41)	−4.25 (−8.14; −0.37) *

^a^ Crude Coefficient; ^b^ Adjusted to age, employment, gender, living area, education, and marital status. * *p* < 0.05.
